# DNMT1 and HDAC2 Cooperate to Facilitate Aberrant Promoter Methylation in Inorganic Phosphate-Induced Endothelial-Mesenchymal Transition

**DOI:** 10.1371/journal.pone.0147816

**Published:** 2016-01-27

**Authors:** Xiaoying Tan, Xingbo Xu, Michael Zeisberg, Elisabeth M. Zeisberg

**Affiliations:** 1 Department of Cardiology and Pneumology, Göttingen University Medical Center, Georg August University, Göttingen, Germany; 2 Department of Nephrology and Rheumatology, Göttingen University Medical Center, Georg August University, Göttingen, Germany; 3 German Center for Cardiovascular Research (DZHK), Göttingen, Germany; University of Bonn, Institut of experimental hematology and transfusion medicine, GERMANY

## Abstract

While phosphorus in the form of inorganic or organic phosphate is critically involved in most cellular functions, high plasma levels of inorganic phosphate levels have emerged as independent risk factor for cardiac fibrosis, cardiovascular morbidity and decreased life-expectancy. While the link of high phosphate and cardiovascular disease is commonly explained by direct cellular effects of phospho-regulatory hormones, we here explored the possibility of inorganic phosphate directly eliciting biological responses in cells. We demonstrate that human coronary endothelial cells (HCAEC) undergo an endothelial-mesenchymal transition (EndMT) when exposed to high phosphate. We further demonstrate that such EndMT is initiated by recruitment of aberrantly phosphorylated DNMT1 to the RASAL1 CpG island promoter by HDAC2, causing aberrant promoter methylation and transcriptional suppression, ultimately leading to increased Ras-GTP activity and activation of common EndMT regulators Twist and Snail. Our studies provide a novel aspect for known adverse effects of high phosphate levels, as eukaryotic cells are commonly believed to have lost phosphate-sensing mechanisms of prokaryotes during evolution, rendering them insensitive to extracellular inorganic orthophosphate. In addition, our studies provide novel insights into the mechanisms underlying specific targeting of select genes in context of fibrogenesis.

## Introduction

Phosphorus in the form of inorganic or organic phosphate is critically involved in most cellular functions including synthesis of DNA/RNA (each nucleotide contains a phosphate group, forming the alternating sugar phosphate backbone of nucleic acids), energy metabolism (as critical constituent of adenosine-tri-phosphate, ATP and nicotinamide adenine dinucleotide, NAD), intracellular signaling (phosphorylation and de-phosphorylation of signaling proteins, constituent of cyclic guanosine monophophosphate, cGMP) and membrane integrity (as constituent of sphingolipids) [1]. While for single-cell organisms access to phosphate from the environment is essential for vital function, humans can store and maintain phosphate at physiological levels through an intricate regulatory system: In humans phosphate homeostasis is determined by balance of intestinal absorption, renal phosphate excretion, release and storage of phosphate from bones [2]. The average healthy adult has approximately 25 Mol of phosphate, with 85% being bound in bone and 15% distributed in soft tissues and extracellular fluids[1]. Principal mediators of phosphate homeostasis include parathyroid hormone (PTH), vitamin D, fibroblast growth factor 23 (FGF23) and Klotho, which maintain circulating phosphate levels within a normal physiological range [3].

Phosphate balance is receiving utmost attention in biomedical research, because elevated serum phosphate levels are associated with decreased life-expectancy and increased cardiovascular morbidity and mortality[4]. Such association is best established in patients with chronic kidney disease in which plasma phosphate levels are elevated due to impaired urinary phosphate secretion [5]. Furthermore, higher plasma phosphate levels (still within “normal” range) in patients with normal kidney function are associated with increased mortality, establishing phosphate as independent risk factor[6, 7].

While the link between increased phosphate serum levels and increased cardiovascular mortality was corroborated by numerous epidemiological studies, the underlying mechanisms are less clear. While several studies demonstrated causal roles of phospho-regulatory hormones FGF23 and Klotho on cardiovascular morbidity[8, 9], high phosphate levels still decreased life-span in FGF23- and Klotho-deficient mice [10–12]. Furthermore, ingestion of high-phosphate meals in healthy volunteers caused transient vascular dysfunction, suggesting direct impact of inorganic phosphate on endothelial cells within two hours, independent of Klotho or FGF23 levels[4].

In context of cardiac morbidities endothelial-mesenchymal transition (EndMT) has emerged as cellular mechanism linking endothelial cells to heart disease[13]. Endothelial-mesenchymal transition (EndMT) is defined as a cellular process whereby endothelial cells transform into a more mesenchymal cell type, associated with elevated expression of mesenchymal marker proteins such as smooth muscle actin (α-SMA) or fibroblast-specific protein-1 (FSP1) and the complementary loss of typical endothelial markers such as CD31 (Pecam-1) and Vascular Endothelial (VE)-cadherin [13, 14]. EndMT was originally discovered during heart development, as a process by which the endocardial endothelial cells of the atrioventricular canal undergo to form the endocardial mesenchymal cushion and later develop into mitral and tricuspid valves[15, 16]. Endocardial fibroelastosis (EFE), a common complication of pre-natal heart defects including hypoplastic left heart syndrome is a direct cause of pathological EndMT as fibroblasts within EFE tissue almost exclusively originate from the endocardium via pathological EndMT[17]. In context of post-natal heart disease, several studies demonstrated that EndMT causally contributes to microvascular rarefication and cardiovascular remodeling and fibrosis [13, 18–22]: While in existing tissues balanced angiogenesis and capillary regression maintain the appropriate relationship between capillary density and oxygen requirements [23], EndMT is being viewed as pathological form of angiogenesis which shifts the balance towards microvascular rarefication [14]. In this regard, EndMT resembles the acquisition of mesenchymal characteristics which are typical of sprouting tip cells in angiogenesis, but differs in regard to formation of functional endothelia, as mesenchymal characteristics are being maintained in EndMT and even further enhanced as becomes evident by excessive production of collagens [14, 24]. In this regard, epigenetic modifications, such as promoter CpG island hypermethylation have been identified as determinant of pathological EndMT in cardiac pathologies, even though the mechanisms underlying such aberrant hypermethylation of specific genes are not yet understood[25].

Here we explored the possibility that increased concentrations of extracellular inorganic phosphate, reminiscent of those encountered by endothelial cells in patients with chronic hyperphosphatemia, could induce EndMT in coronary endothelial cells, independent of phospho-regulatory hormones. We demonstrate that exposure to 3mM inorganic phosphate causes EndMT associated with *RASAL1* promoter CpG island promoter methylation. We further demonstrate that *RASAL1* promoter methylation in response to high phosphate is dependent on increased phosphate influx, subsequent phosphorylation and activation of the DNA methyltransferase DNMT1, which is recruited to the *RASAL1* promoter by HDAC2.

## Materials and Methods

### Cell culture and immunofluorescence staining

HCAECs (purchased from Genlantis, San Diego, USA) were maintained in HCAEC culture medium (Genlantis, San Diego, USA) and were passaged according to the manufacture’s instruction. Cells were serum starved with endothelial basic medium for 24 hours prior to any treatment. Unless noted, all experiments were performed in basic medium condition which contains 3mM Pi and concentrations were listed in the figures, and basic medium which contains1mM phosphate was served used as treated group. Added Pi is in the form of NaPO4, pH 7.4 (Sigma-Aldirch). To inhibit EndMT, 50μM Farnesylthiosalicylic Acid (Sigma- Aldirch, Germany) or 30 μM phosphonoformic acid (Sigma- Aldirch, Germany) was pre-incubated with cells for 30min before addition of phosphate. For TGFβ-induced EndMT, cells were treated with TGFβ1 at 10ng/ml for 4 days after starvation for 24 hours. For immunofluorescence staining, cells were seeded onto 4- chamber culture slides (BD Falcon). After phosphate treatment, the cells were fixed with ice cold methanol/ acetone(1:1) at -20°C for 10min followed by permeabilization with 0.1% Triton X-100 in PBS and then blocked with 1%BSA in PBS for 30min at room temperature. The cells were subjected to immunofluorescence staining with primary antibody CD31 (1:100, Dako) or aSMA(1:100, Abcam) or S100A4 (1:100, Abcam) for 2 h at room temperature. The cells were washed with cold PBS, and incubated with Alexa 568-labeled anti-rabbit (1:300) and Alexa 488-labeled anti-mouse (1:300) secondary antibodies (Life technologies) at room temperature for 1 h. The cells were examined by fluorescence microscopy Zeiss Axiovert200 and AxioVision 3.0 software. The acquired imaged were processed using Photoshop CS3 software.

### Cell proliferation assays

Cell viability was performed using XTT (2,3-Bis-(2-Methoxy-4-Nitro-5-Sulfophenyl)-2H-Tetrazolium-5-Carboxanilide) assay according to manufacturer's instruction (Promega, Madison, WI). 4×10^3^ cells /100 μL per well were plated in 96 well plates. After incubation over- night, the cells were treated with 0, 1, 2, 3 mM Pi (1, 2, 3, and 4mM final) for 24, 48 or 72 hours respectively. The absorbance was measured 1 hour after incubation with XTT assay reagent on a Synergy 2 Multi-Mode Reader (BioTeck). The results are from 3 replicates/ treatment and 3 independent experiments.

### Co- Immunoprecipitation assay

HCAEC cells treated with phosphate or with TGF-β were used to prepare the protein extract using RIPA buffer (Millipore). The protein extracts were pre- cleaned with protein A/G agrose beads for 3 h at 4°C. The supernatants were then incubated with 5μl of antibodies overnight at 4°C on a roller. Next day, beads were washed and the immunoprecipitated protein complexes were eluted using laemmli buffer, separated on 4–12% SDS-PAGE and subjected to Western blot analysis with indicated antibodies.

### Chromatin immunoprecipitation (ChIP)

ChIP assays were performed with OneDay ChIP kit (Diagenode) and followed manufacture’s protocols^14^. Briefly, cells were cross- linked and then lysed with shearing kit (Diagenode) followed by sonication (Misonix). The sheared chromatin was immunoprecipitated with 5 μg of HDAC2 (Cell signaling) or DNMT1 antibody (Thermo scientific) or with IgG as a negative control (Diagenode), processed with the Diagenode OneDay ChIP protocol. Quantitative analysis of the immunoprecipitated DNA was analyzed by StepOne Real-Time System (Applied Biosystems), using primers (sequences are listed in Table 1) flanking human *RASAL1* promoter region and one pair of primers flanking exon 8 as negative control. The ChIP-qPCR data were analyzed using ΔCt method in which the immunoprecipitated sample Ct value was normalized with the input DNA Ct value and the percentage of precipitation was calculated using the following formula (%Input = 2^-(Ct Iped—Ct Input) x dilution factor x 100%).

**Table 1:** The sequence of primers used for ChIP assay.

name	sequence	supplier
hRasal1_ChipF:	GCCACCTCACCAGGAGCC AGCGGCC	Eurofins MWG Operon
hRasal1_ChipR:	CTACCGGCACCCCAGTCATGC GC	Eurofins MWG Operon
hRasal1_ngF:	TCCCACTCACAGACCACTTC	Eurofins MWG Operon
hRasal1_ngR:	ACATCCACCCTTCTGAGAGC	Eurofins MWG Operon

### Mass spectrometry analysis

The HCAEC cell protein extracts were immunoprecipitated using anti-DNMT1(Thermo Scientific) antibody and the resulting protein complex was resolved on 4–12% SDS-PAGE and stained using coomassie blue staining solution. The indicated protein bands were excised out of the gel, the proteins reduced and carbamidomethylated, in-gel digested with trypsin and analyzed by hybrid quadrupole-orbitrap electrospray mass spectrometer (Thermo Scientific Q Exactive). Combined mass lists from MS/MS spectra were used for a database search in the NCBInr Protein database.

### Methylated DNA immunoprecipitation

MeDIP assay was performed according to the factory’s manual. Briefly, methylated DNA was first captured using Methylamp Methylated DNA capture Kit (Epigentek, Farmingdale, USA) then sonicated into small fragments. 1.0μg of sonicated DNA as input was used in each antibody coated well and incubated at room temperature for 2 hours on a horizontal shaker. The captured DNA was eluted with proteinase K at 65°C for 1 hour. DNA was purified using the column and adjusted to a final volume of 100μl with nuclease-free water [26].

### Protein extraction and Western blotting

HCAEC cell protein was extracted using NP40 lysis buffer (Life technologies), containing EDTA- free protease inhibitor cocktail (Roche) and phosphatase inhibitor (Sigma-Aldrich). Protein samples were resolved on 4–12% SDS-PAGE then transferred onto nitrocellulose membrane (Amersham Biosciences). The membrane was first blocked with 5% dry milk in TBST (TBS pH7.2, 0.1% Tween-20), then was incubated with primary antibodies (detail and dilution factor were listed in Table 2) at 4°C overnight. On the second day, the membrane was washed 3 times with 2% dry milk in TBST, then was incubated with secondary HRP-conjugated antibodies (Cell signaling), and signals were detected using a chemiluminescent kit (Cell signaling).

**Table 2:** The list of antibodies used for Western blotting.

Antibody	Product code	Dilution	Company
p-DNMT1	ZYE1107W	1:1000	Bioss
DNMT1	MA5-16169	1:1000	Pierce
HDAC2	ab16032	1:1000	Abcam
RASAL1	46–269	1:1000	ProSci
a-Tubulin	Sig T5168	1:5000	Sigma-Aldrich

### RNA extraction and real-time PCR

Total RNA was extracted from cells using PureLink RNA kit (Life Technologies) according to company's instruction. 200ng of total RNA was digested with DNase I (Sigma-Aldrich) and used for cDNA synthesis using the SuperScriptII system (Life Technologies). Quantitative real-time PCR analysis was performed with Fast SYBR Master Mix (Life technologies) and run on a StepOne Plus realtime PCR system (Life technologies) with the real-time PCR primers (sequence listed in Table 3). Measurements were standardized to the GAPDH reaction using delta delta Ct methods [27].

**Table 3:** The sequences of primers used for real-time PCR.

Gene	Sequence	Supplier
S100A4	F: TCTTTCTTGGTTTGATCCTGACTR: AGTTCTGACTTGTTGAGCTTGA	Primer designSouthampton, UK
VE-cadherin	F: CAGCCCAAAGTGTGTGAGAAR: CAGCCCAAAGTGTGTGAGAA	Eurofins MWG Operon(Niessen et al., 2008[28])
GAPDH	undisclosed	Primer designSouthampton, UK
SLUG	F: ACTCCGAAGCCAAATGACAAR: CTCTCTCTGTGGGTGTGTGT	Primer designSouthampton, UK
SMAD2	F: GGAGCAGAATACCGAAGGCAR: CTTGAGCAACGCACTGAAGG	Eurofins MWG Operon(Yu et al., 2009[29])
SNAIL	F:GGCAATTTAACAATGTCTGAAAAGGR:GAATAGTTCTGGGAGACACATCG	Primer designSouthampton, UK
DNMT1	F: TTCTGTTAAGCTGTCTCTTTCCAR: TGCTGAAGCCTCCGAGAT	Primer designSouthampton, UK
DNMT3a	F: ATAGATCCCGGTGTTGAGCCR: ACCCAGCGCAGAAGCAG	Primer designSouthampton, UK
DNMT3b	F: GTTTCCCGGAAGAGCTTTGR: GGGAGGTGTCCAGTCTGCTA	Primer designSouthampton, UK
DNMT3l	F: GCCGTACACAAGATCGAAGGR: GTTCTGACCCGGGACAACT	Primer designSouthampton, UK
RASAL1	F: CGTGCTGGATGAGGACACTGR: TCCCTGCTCAGCGAGATCTT	Primer designSouthampton, UK

### Statistical analysis

All qPCR data for RNA expression analysis (two or more biological replicates) were calculated using the ΔΔCT method. Student t-test (GraphPad Prism 5.1) was used to obtain calculations of statistical significance.

## Results

**Fig 1:**
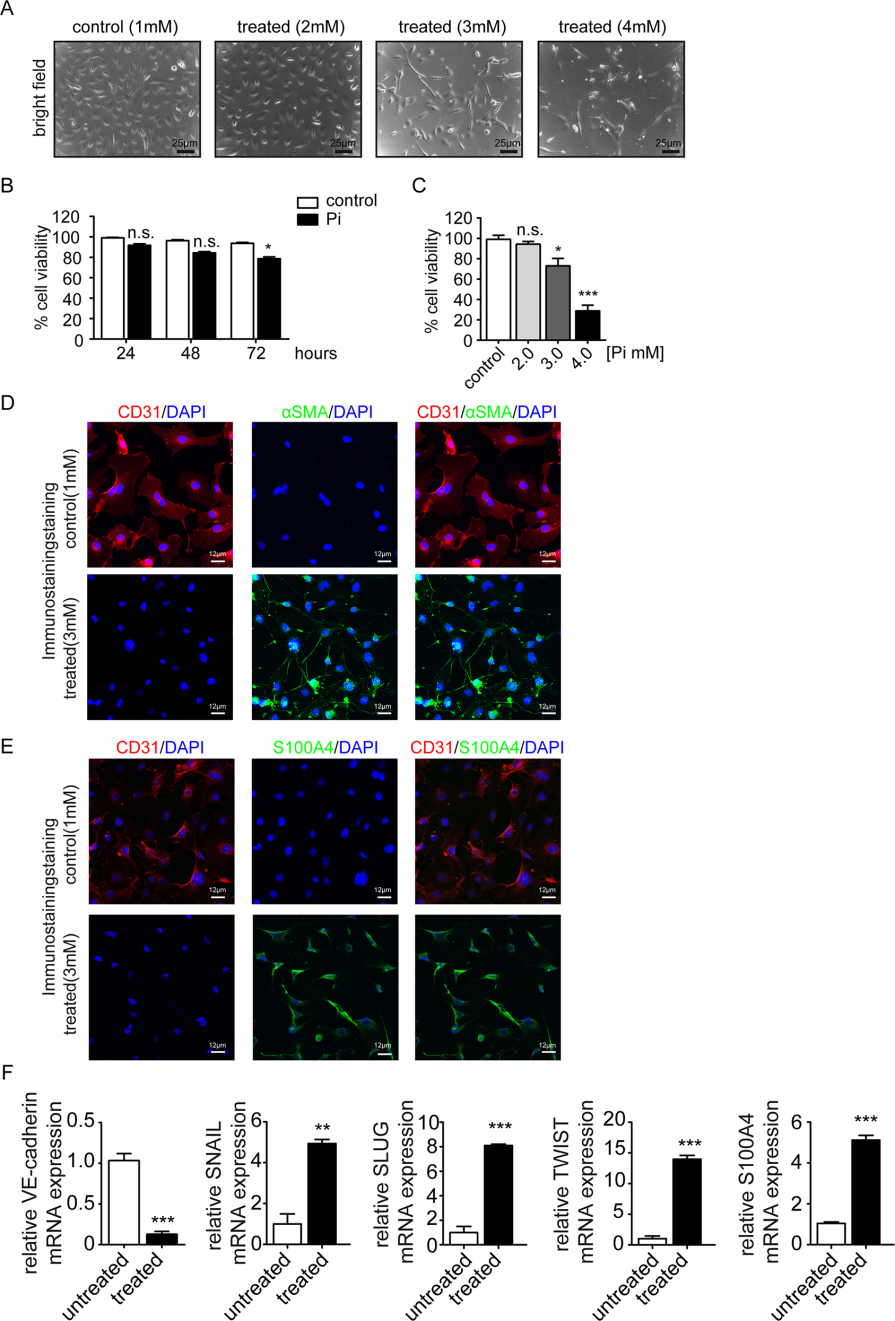
High inorganic phosphate triggers endothelial to mesenchymal transition. (A) bright-field images showing the morphology changes of human coronary artery endothelial cells (HCAEC) incubated for 3 days with Pi (1mM to 4mM) compared with control cells. Scale bars 25 μm. (B) Cell viability was measured by XTT assay in HCAEC cells treated with 3mM inorganic phosphate (Pi) for 24, 48, and 72hours. (C) Cell viability was measured by XTT assay in HCAEC cells after treated with various concentration of inorganic phosphate (1mM to 4mM) for 3 days. Results shown are mean±s.e.m. for three independent experiments. (D- E) Representative immunofluorescence images showing CD31 (red) and α-SMA/ S100A4 (green) staining in control (upper panel) and high Pi treated cells (lower panel); nuclei were counterstained with DAPI (blue). Scale bars 10 μm. (F) qRT-PCR data showing the mRNA expression levels of endothelial marker VE-cadherin, EndMT transcriptional factors (SNAIL, SLUG, and TWIST) and S100A4 in control and high Pi treated cells. Results were normalized to reference gene GAPDH (expression is presented as means ± s.d., n = 3 independent experiments, **P<0.01, ***P<0.001).

To elucidate possible impact of increased extracellular concentration of inorganic phosphate (Pi) on endothelial cells, we utilized human coronary aortic endothelial cells (HCAEC), which are commercially available, and which have been extensively characterized as a population of homogeneous endothelial cells in previous studies[14]. HCAEAC were exposed to Pi from 1mM (within range of normal phosphate concentration in humans) to 4mM (range of increased phosphate encountered in patients is up to 3mM). We observed that HCAEC exposed to standard media supplemented with 3mM phosphate decreased in cell number and acquired a spindle-shaped morphology, reminiscent of phenotypic changes typically associated with endothelial-mesenchymal transition (EndMT) (Fig 1A). In addition, whereas 4 mM dramatically reduced cell survival, 3mM only had moderate influence on cell viability (Fig 1B and 1C). To further corroborate this observation we performed immunofluorescence double-labeling experiments using antibodies specific for CD31 and α-smooth muscle actin (α-SMA)/ fibroblast-specific protein (S100A4) and we observed that acquisition of spindle-shaped morphology in response to 3mM phosphate was associated with loss of the endothelial marker CD31 and *de novo* expression of the myofibroblast/ fibroblast marker α-SMA/ S100A4, typical of EndMT (Fig 1D and 1E). Such phenotypic changes were associated with decreased endothelial cell marker VE-cadherin expression and increased expression of fibroblast cell marker S100A4 and the three transcription factors SNAIL, SLUG and TWIST, which are typically considered as master-regulators of transcriptional program associated with EndMT phenotypic conversions (Fig 1F).

**Fig 2:**
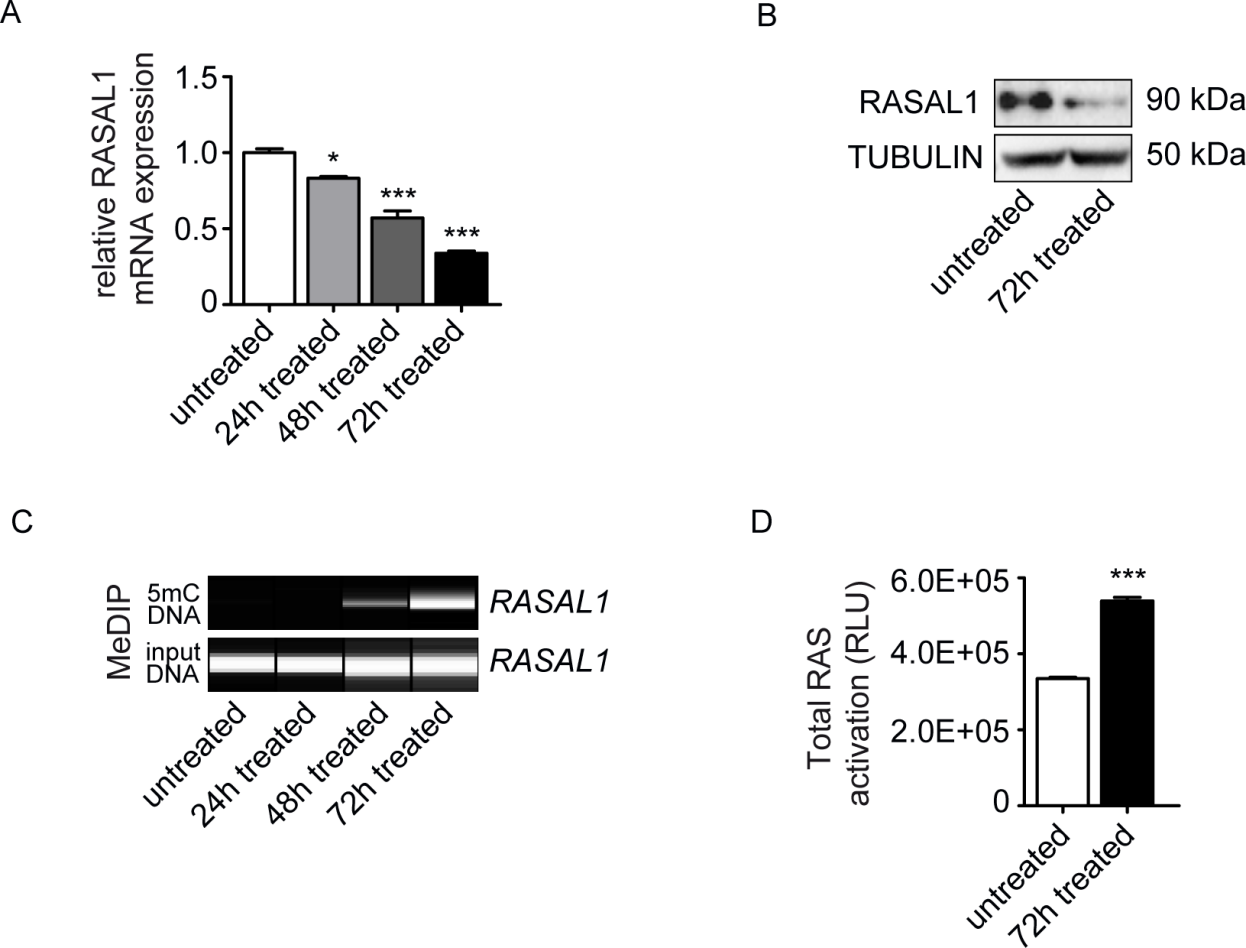
High Pi induces hyper-activity of Ras through inhibiting RASAL1 expression. (A) Decreasing mRNA expression levels of RASAL1 upon the time of high Pi treatment after 24, 48 and 72 hours. Results were normalized to reference gene GAPDH (expression is presented as means ± s.d., n = 3 independent experiments, *P<0.05, **P<0.01, ***P<0.001). (B) Western blot confirming the decreased protein expression of RASAL1 in Pi treated cells. (C) MeDIP result showing the methylated promoter of RASAL1 along the treatment time, correlated with the reduced expression of RASAL1 in both mRNA and protein level. (D) Ras activity was measured by ELISA assay, untreated cells served as controls. Pi- treated cells showed the hyper-activation of total Ras.

Based on our previous studies in which we had identified aberrant promoter CpG island methylation of *RASAL1* (a member of the Ras-GAP like proteins which serve as negative regulators of Ras-GTP signaling) and its subsequent transcriptional suppression as a crucial event in the EndMT involving HCAEC [25], we next explored if this pathway was also involved in the EndMT observed upon culture in media containing high phosphate levels. Analysis by quantitative real-time PCR (qRT-PCR) revealed RASAL1 expression levels decreased gradually over 72 hours when HCAEC were cultured in media containing 3mM phosphate as compared to cells cultured in 1mM phosphate (Fig 2A). Such decreased RASAL1 mRNA expression correlated with decreased RASAL1 protein levels (Fig 2B) and also with increased promoter CpG island methylation of the proximal *RASAL1* promoter (Fig 2C), which we had identified as being responsible for decreased RASAL1 expression in previous studies. Decreased levels of the Ras-GTP inhibitor RASAL1 in HCAEC upon culture for 72 hours in 3mM phosphate corresponded with increased intrinsic Ras-GTP activity (Fig 2D). In summary, we observed that presence of 3mM inorganic phosphate (reminiscent of serum phosphate levels observed in patients with hyperphosphatemia) induces EndMT in cultured HCAEC and that this phenotypic conversion was associated with aberrant methylation of the Ras-GTP inhibitor *RASAL1*, an event known to facilitate EndMT in HCAEC.

**Fig 3:**
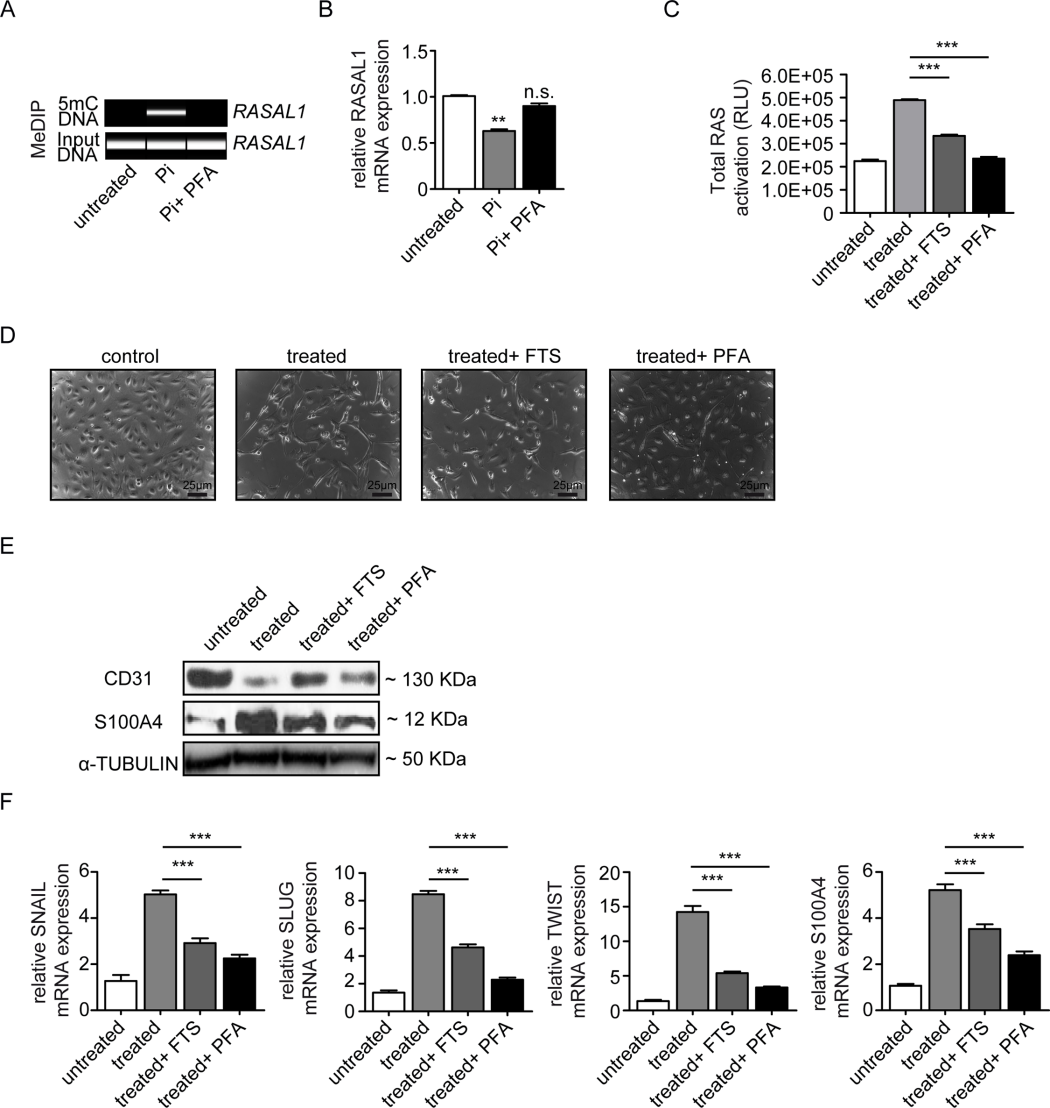
phosphate transporter inhibitor inhibits endothelial to mesenchymal transition through restored RASAL1 expression. Cells were treated with Pi 3mM for 72 hours. (A) MeDIP result showing the demethylated promoter of RASAL1 by using PFA, correlated with restored mRNA expression of RASAL1. (B) qPCR analysis showed the restored RASAL1 mRNA expression levels by using phosphate transporter inhibitor (PFA). Results were normalized to reference gene GAPDH (expression is presented as means ± s.d., n = 3 independent experiments, **P<0.01). (C) ELISA assay indicating the normalization of total Ras with combination of PFA or RAS inhibitor farnesylthiosalicylic acid (FTS). (D) bright-field images showing the morphology change of HCAEC cells cultured under normal control condition, high Pi conditions, and Pi combined with FTS or PFA. Scale bars 25 μm. (E) Western blot analysis showing the expression of endothelial cell marker CD31 and fibroblast cell marker S100A4 in HCAEC cells exposed to normal control condition, high Pi conditions, and Pi combined with FTS or PFA (F) qRT-PCR data showing the mRNA expression levels of EndMT transcriptional factors (SNAIL, SLUG, and TWIST) and FSP1 in HCAEC cells under four conditions indicated above. Results were normalized to reference gene GAPDH (expression is presented as means ± s.d., n = 3 independent experiments, ***P<0.001).

We next aimed to gain further insights into a possible causal link of high phosphate, *RASAL1* methylation and EndMT. For this purpose we added 30 μM phosphonoformic acid (PFA), an inhibitor of sodium-dependent phosphate transporters[30]. Inhibition of phosphate influx prevented RASAL1 promoter methylation and subsequent transcriptional repression in response to high phosphate (Fig 3A and 3B). Furthermore, addition of PFA to culture media containing high phosphate inhibited Ras-GTP activity, mimicking the effect of the Ras-inhibitor Farnesylthiosalicylic Acid (Fig 3C). Both FTS and PFA inhibited EndMT in response to high phosphate was determined by cell morphology (Fig 3D) and analysis of endothelial cell marker CD31 and fibroblast cell marker S100A4 and EndMT transcriptional markers SNAIL, SLUG and TWIST (Fig 3E and 3F). In summary our studies demonstrated that EndMT in response to high phosphate is causally linked to aberrant CpG promoter methylation of *RASAL1* and subsequently increased intrinsic Ras-GTP signaling activity and Twist/Snail activation. Our studies further revealed that such effect is dependent on influx of inorganic polyphosphate.

**Fig 4:**
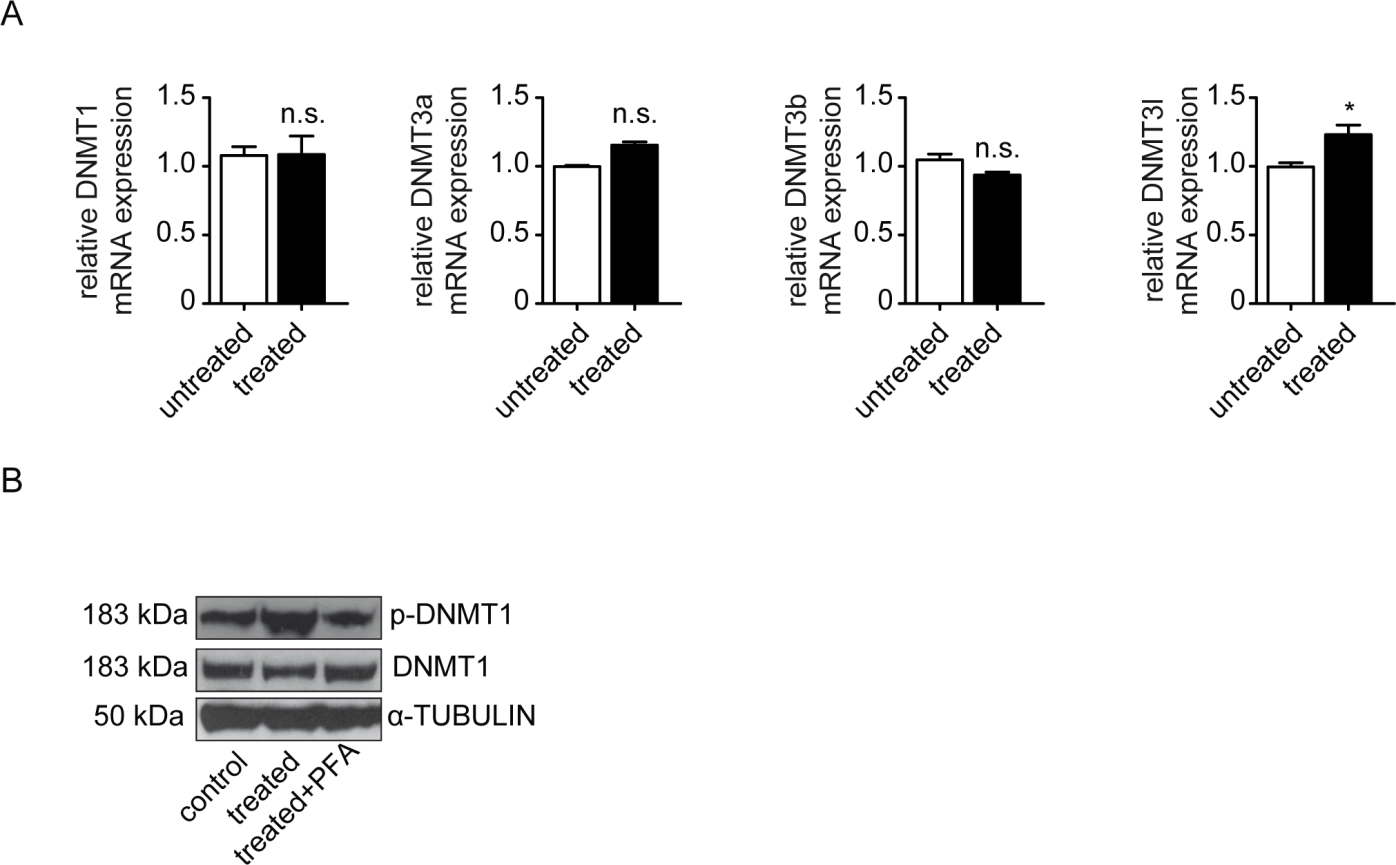
High Pi influences HCAEC mediated by increased DNMT1 activity. Cells were treated with Pi 3mM for 72 hours. (A) qRT-PCR data showing the mRNA expression of DNA methyltransferases (DNMT1, DNMT3a, DNMT3b and DNMT3l) in control and Pi treated cells. (B) Western blot demonstrating the enhanced protein level in phosphorylated DNMT1-ser154 stimulated by Pi treatment, which was inhibited by phosphate transporter inhibitor Phosphonoformic Acid (PFA), whereas total DNMT1 was not affected.

We next aimed to gain additional insights into mechanisms linking high extracellular phosphate levels to aberrant promoter methylation of *RASAL1*. We first analyzed expression levels of the three enzymes which possess DNA methylating activities in principle, the DNA-methyltransferases DNMT1, DNMT3a and DNMT3b by qRT-PCR. However, neither of the DNMT expression levels changed in response to high phosphate (Fig 4A). Based on a recent study which had revealed that activity of DNMTs is not only regulated at the transcriptional levels but also through possible phosphorylation of a Ser154residue in DNMT1 [31], we next performed immunoblot analysis of HCAEC total cell lysates using antibodies specific for either total DNMT1 or phosphorylated DNMT1. Such analysis revealed that upon culture in high phosphate media DNMT1 phosphorylation increased substantially (Fig 4B), an observation which is completely in line with previous reports that identified DNMT1 as the enzyme responsible for aberrant *RASAL1* promoter CpG island methylation[26, 32]. In summary, our data suggested that exposure to 3mM inorganic phosphate induces EndMT in HCAEC via a mechanism, which involves hypermethylation of the RASAL1 CpG island promoter by the methyltransferase DNMT1. Because this pathway is also critically involved in TGFβ1-induced EndMT, these findings suggested that DNMT1-mediated RASAL1 hypermethylation is part of the EndMT master-program, raising further interest in its underlying mechanisms.

**Fig 5:**
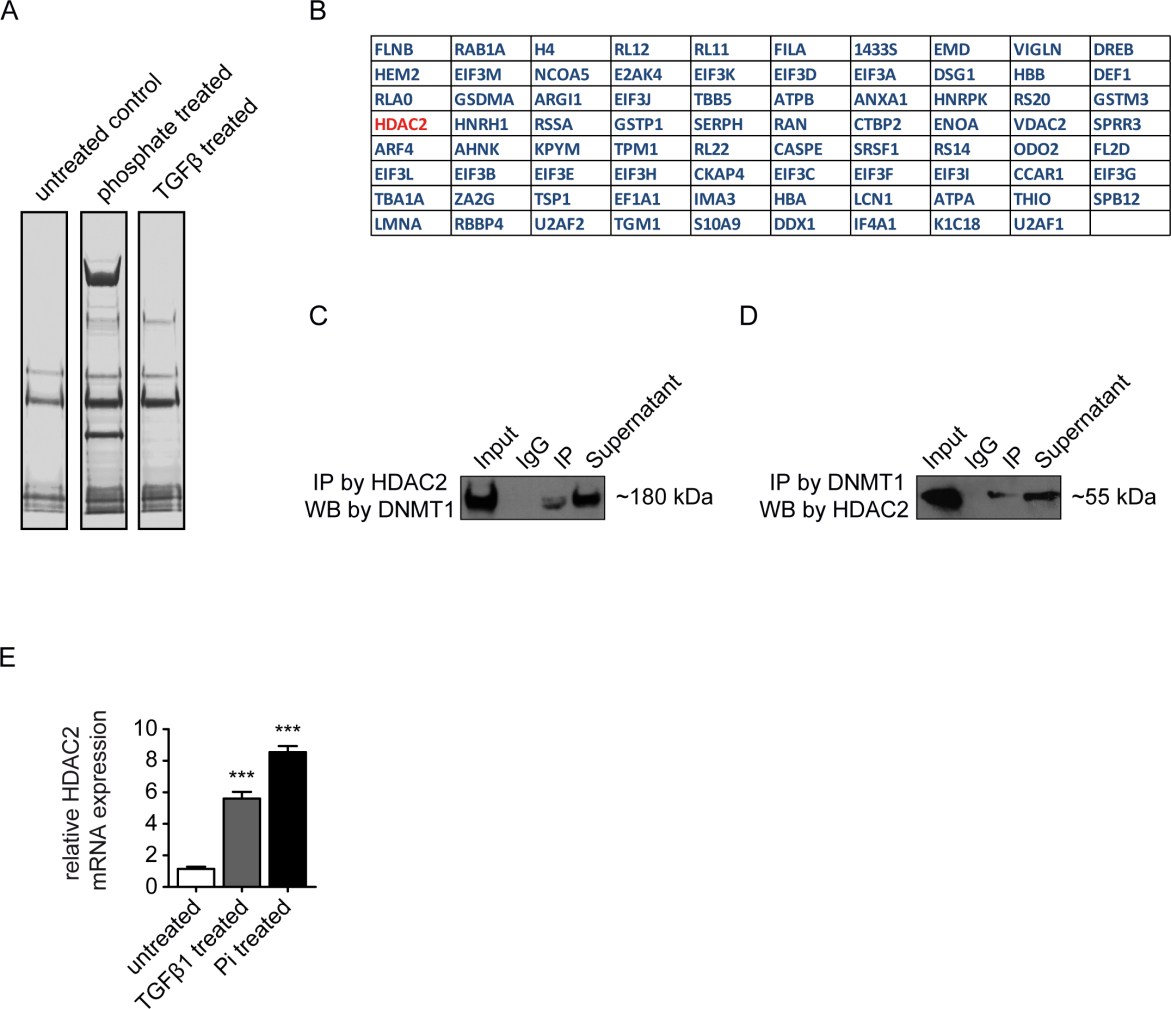
Mass spectrometry (MS) based proteomics coupled with co-immunoprecipitation for Pi treated and TGF-β1 cells. (A) Protein complex from phosphate (3mM for 72 hours) and TGFβ (10ng/ml TGFβ1 for 4 days) treated cells were immunoprecipitated by DNMT1 antibody and resolved in SDS-PAGE gel which stained by Coomassie blue. Untreated cells were used as control. (B) A list of protein candidates identified through Mass spectrometry analysis showed the commonly enriched proteins through DNMT1 immunoprecipitation from Pi and TGF-β1 treated cells. (C-D) Confirmation of the interaction between DNMT1 and HDAC, the protein complex was immunoprecipitated by (C) HDAC2 antibody and detected by DNMT1 antibody or was immunoprecipitated by (D) DNMT1 antibody and detected by HDAC2 antibody. (E) qRT-PCR analysis showed the upregulated mRNA expression of HDAC2 in both Pi and TGF-β1 treated cells. Results were normalized to reference gene GAPDH (expression is presented as means ± s.d., n = 3 independent experiments, ***P<0.001).

While enzymatic activity of DNMT1 to methylate select genes in context of pathologies is well-documented[32–34], mechanisms which underlie such target selectivity are not yet completely understood. Based on the thinking that target selectivity of DNMT1 is determined through interaction with one or several chromatin-binding proteins, we next performed co-immunoprecipitation comparing lysates from cells maintained in serum free-media containing normal phosphate, in serum-free media containing high phosphate or in serum free media containing normal phosphate which were supplemented with TGFβ1, using antibodies to DNMT1 and analyzed pulled-down proteins by mass spectrometry (Fig 5A). Because the number of proteins identified in TGFβ-treated versus untreated and phosphate treated versus untreated individually was so high (451 candidates for TGFβ-treated cells and 751 for phosphate-treated cells), we decided to focus on the commonly bound proteins in TGFβ and phosphate treated cells (Fig 5B). Among the top 10 differentially captured proteins upon both exposure to high phosphate or TGFβ1 was the histone deacetylase HDAC2 (Fig 5B, 5C and 5D). Increased HDAC2 expression in HCAEC in response to either TGFβ1 or high phosphate further provided evidence for involvement of HDAC2 in aberrant *RASAL1* CpG island promoter methylation by DNMT1 and we hypothesized that HDAC2 is possibly involved in recruiting DNMT1 to the CpG island within the *RASAL1* promoter (Fig 5E).

**Fig 6:**
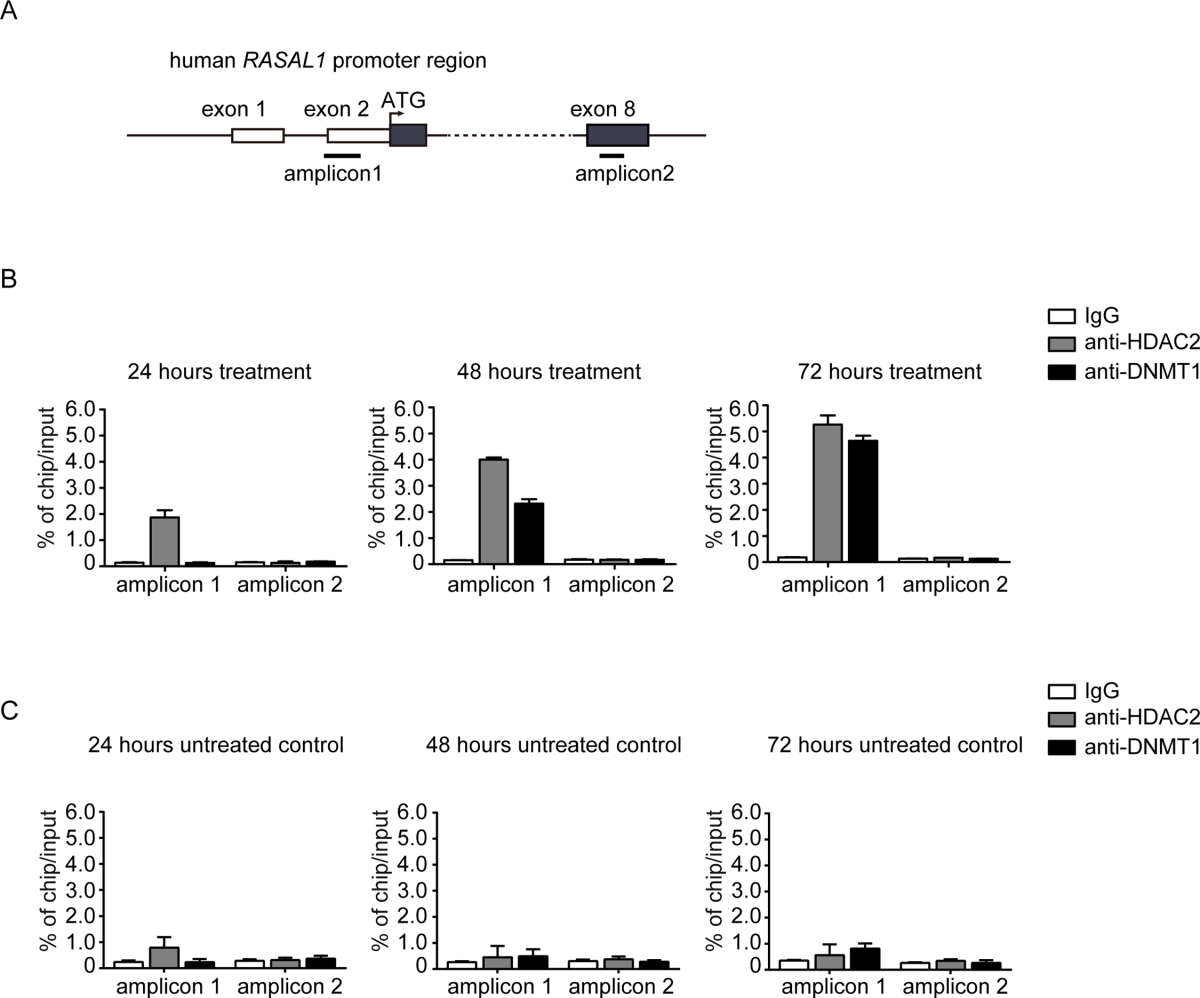
Pi inhibits RASAL 1 expression through direct binding to its promoter. (A) Simplified schematic showing the human RASAL1 promoter along with exons (black boxes), translational start site (black arrow), locations of RASAL1 chip primer for amplicon1 and amplicon2, and amplicon2 primers serve as negative control chip primer. (B-C) The binding properties of HDAC2 and DNMT1 to the RASAL1 promoter region were analyzed by chromatin immunoprecipitation (ChIP) assay and detected by qRT-PCR in Pi treated (B) or in control cells (C). IgG purified from the same species serve as negative control for ChIP (expression are presented as means ± s.d., n = 3 independent experiments, **P<0.01, ***P<0.001, n.s. no significance).

To test this hypothesis we performed time-course ChIP-qPCR analysis using antibodies to HDAC2 or DNMT1 and primers specific for CpG island flanking the ATG start site of RASAL1 or exon 8 after exposure to high phosphate for 24, 48 or 72 hours (Fig 6A). This analysis revealed that HDAC2 binding to the *RASAL1* ATG promoter CpG island (observed after 24 hours), precedes recruitment of DNMT1 to the *RASAL1* promoter, which could only be detected after 48 hours of cultivation in high phosphate (Fig 6B and 6C), coinciding with onset of aberrant *RASAL1* CpG methylation observed under these conditions (compare to Fig 2C). In summary, our findings suggest that high phosphate reflective of systemic levels encountered in patients with chronic kidney disease induce EndMT in cultured HCAEC through a pathway involving methylation of the *RASAL1* CpG island promoter and subsequent transcriptional silencing of the Ras-GTP suppressor RASAL1 and increased intrinsic Ras-GTP activity. Furthermore, our data suggests that such aberrant RASAL1 promoter methylation is mediated by methyl transferase DNMT1 and that HDAC2 is involved in the process of recruiting DNMT1 towards the *RASAL1* promoter.

## Discussion

Here we provide evidence that high extracellular phosphate directly induces EndMT in cultured HCAEC, independent of phospho-regulatory hormones such as FGF23 and Klotho. Even though phosphate sensing receptors on mammalian cells are not yet known, direct induction of biological responses by inorganic phosphate is not without precedent as previous studies also documented direct responses of cultured osteoblasts when phosphate levels within culture media were increased from 1mM to 3mM [35, 36].

**Fig 7:**
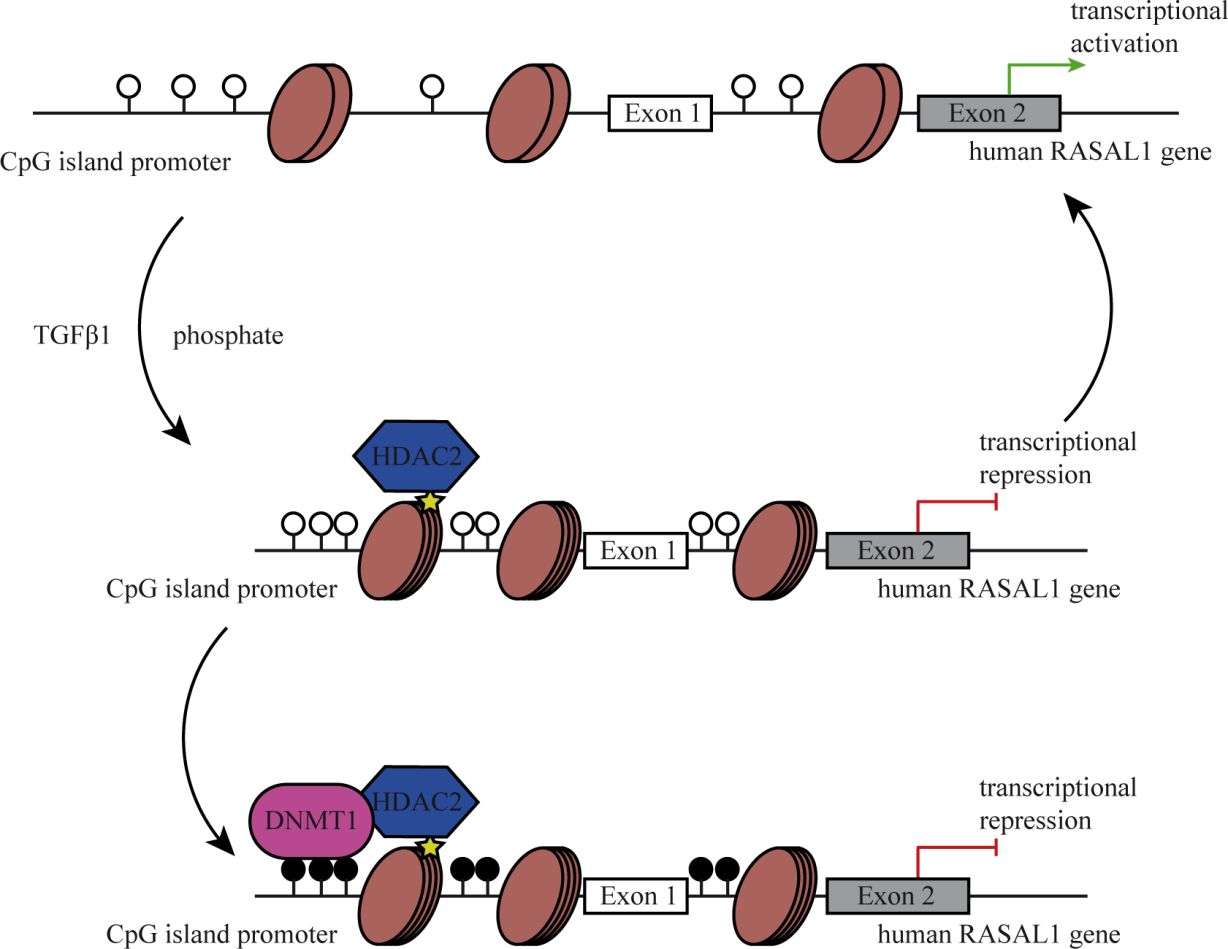
Schematic representation of epigenetic regulating RASAL1 promoter by HDAC2 and DNMT1. In the physiological conditions, the CpG islands located in RASAL1 promoter are unmethylated (top panel) as indicated by open circles, and RASAL1 is transcriptional active. Under pathological conditions, initially when endothelial cells are exposed to stimulus, such as TGFβ1 or high concentration of Pi resulting in RASAL1 silencing through condensed chromatin structure at RASAL1 promoter mediated by HDAC2. While the RASAL1 promoter remains unmethylated (middle panel) and RASAL1 is transiently silenced. When the cells are continuously exposed to the stimulus, the CpG islands located in RASAL1 promoter are methylated (indicated by filled circles) by DNMT1 recruited through the interaction with HDAC2. Therefore, RASAL1 is permanently silenced due to promoter hypermethylation (lower panel).

Our studies reveal a mechanism through which influx of inorganic phosphate causes recruitment of HDAC2 toward the RASAL1 promoter, which in turn recruits phosphorylated, activated DNMT1 to facilitate RASAL1 promoter CpG island methylation and transcriptional repression (Fig 7). Ensuing increased Ras-GTP activity results in activation of transcription factors Snail, Twist and Slug, resulting in EndMT of HCAEC. This observation is in line with previous reports, demonstrating that environmental factors linked to progression of cardiac remodeling and fibrosis such as growth factors TGFβ [13, 37] and CTGF [38], hypoxia [14], inflammatory TLR5-mediated signaling responses [39] and asymmetric dimethylarginine (ADMA)[40] all induce EndMT associated with Snail and/or Twist activation. While this suggests that EndMT might be reflection of a common cellular stress-response program, individual signaling pathways to activate Snail and/or Twist may be different and require further studies. Furthermore, several studies demonstrated that fibrotic EndMT is associated with epigenetic modifications such as aberrant Promoter methylation of select genes (such as RASAL1 or BMP7), histone modifications, or altered microRNA profiles[17, 25, 41–43]. In this regard, our study for the first time demonstrates that the different epigenetic mechanisms work cooperatively in context of EndMT, possibly to stabilize the mesenchymal phenotype.

In this regard, our study provides evidence for a causal link of HDAC2 and phosphorylated DNMT1 in RASAL1 promoter methylation and transcriptional silencing. Of note, we have shown in previous studies that knockdown of DNMT1 prevented RASAL1 promoter methylation[32]. Additionally, Rountree et al. demonstrated that DNMT1 forms a complex with HDAC2 to inhibit transcription of methylated genes in heterochromatin[44]. While this previous study did not perform time course analysis, the two proposed mechanisms do not exclude another and it is conceptually attractive to speculate that both mechanisms cooperate (recruitment of DNMT1 by HDAC2 to facilitate de novo methylation and then cooperation to suppress transcription of the methylated gene). Further experiments will have to be performed, including knockdown of HDAC2, to address these mechanisms in detail.

Our studies demonstrate that endothelial cells respond to high extracellular inorganic by increased phosphate intake, and that such increased phosphate influx causes cell death and ultimately EndMT. This is in line with previous studies by our group, which demonstrate that endothelial cells treated with serum from patients with chronic kidney disease undergo increased apoptosis and ultimately EndMT[40]. We are aware, however, that the mechanistic links between phosphate influx and DNMT1 phosphorylation and HDAC2 recruitment are entirely unclear. Nevertheless, direct biological impact of inorganic phosphate on endothelial cells adds a new twist in context of the link of high plasma phosphate and increased mortality and the role of an inorganic anion as signaling molecule should be further explored.
